# Fabrication of Large-Area Silicon Spherical Microlens Arrays by Thermal Reflow and ICP Etching

**DOI:** 10.3390/mi15040460

**Published:** 2024-03-29

**Authors:** Yu Wu, Xianshan Dong, Xuefang Wang, Junfeng Xiao, Quanquan Sun, Lifeng Shen, Jie Lan, Zhenfeng Shen, Jianfeng Xu, Yuqingyun Du

**Affiliations:** 1 State Key Laboratory of Intelligent Manufacturing Equipment and Technology, School of Mechanical Science and Engineering, Huazhong University of Science and Technology, Wuhan 430074, China; mse_wuyu@hust.edu.cn (Y.W.); jfxu@hust.edu.cn (J.X.); dyqy@hust.edu.cn (Y.D.); 2 Science and Technology on Reliability Physics and Application Technology of Electronic Component Laboratory, Guangzhou 511370, China; dongxs@pku.edu.cn; 3 Shanghai Aerospace Control Technology Institute, Shanghai 201109, Chinafdchrist@126.com (L.S.); lan_jier@163.com (J.L.); little_fenger@sina.com (Z.S.)

**Keywords:** microlens array, thermal reflow process, ICP etching, etching selectivity

## Abstract

In this paper, we proposed an efficient and high-precision process for fabricating large-area microlens arrays using thermal reflow combined with ICP etching. When the temperature rises above the glass transition temperature, the polymer cylinder will reflow into a smooth hemisphere due to the surface tension effect. The dimensional differences generated after reflow can be corrected using etching selectivity in the following ICP etching process, which transfers the microstructure on the photoresist to the substrate. The volume variation before and after reflow, as well as the effect of etching selectivity using process parameters, such as RF power and gas flow, were explored. Due to the surface tension effect and the simultaneous molding of all microlens units, machining a 3.84 × 3.84 mm^2^ silicon microlens array required only 3 min of reflow and 15 min of ICP etching with an extremely low average surface roughness Sa of 1.2 nm.

## 1. Introduction

Traditional optical lenses have the disadvantages of being large in size and heavy in weight, which do not satisfy the development trend of miniaturization and lightweighting for optical systems. Microlens arrays (MLAs) have emerged as an alternative to large aperture optical lenses and are composed of numerous tiny micron-sized lens units arranged in a specific layout. MLAs can provide excellent optical functions, including illumination, collimation, focusing, imaging, and light redistribution, coupled with significantly reduced mass and volume compared to conventional lenses, enabling great application potential across multiple fields, such as imaging systems [1,2,3,4,5], optical communication [6,7,8], and sensors [9,10].

The fabrication processes of microlens arrays include ultra-precision machining technologies [11,12,13,14,15], laser direct writing [16,17,18], grayscale lithography [19,20], nanoimprint lithography [21,22,23,24], and thermal reflow processes [25,26,27,28]. Ultra-precision machining technologies, such as single point diamond turning (SPDT), are capable of fabricating microstructures with complex morphology and excellent precision. However, continuous cutting for an excessive duration will affect machine tool accuracy and significantly damages the diamond tool, resulting in poor uniformity of the processed microstructures. Yan and Makaida [29] studied a tool-servo driven segmented turning method to fabricate silicon concave microlens arrays. The intermittent contact between the tool and the workpiece can effectively limit tool wear, achieving a surface roughness of 5 nm, but the machining area needs to be further improved. Laser direct writing technology utilizes optical systems to focus a laser beam onto the surface of the workpiece, thus melting and vaporizing the material to fabricate micro/nano structures. Hua et al. [30] used a femtosecond laser with a wavelength of λ=343 nm and a pulse duration of $t_{p}=280\;\mathrm{fs}$ to directly fabricate a convex MLA on silicon with a surface roughness of below 3 nm. Femtosecond laser technology works directly on the target material without additional masks, but it is not suitable for machining large-area microstructures due to the low processing efficiency caused by its inherent characteristic of removing material point by point. The laser used by Hua had a material removal rate of 120 μm^3^/s, and it took 1 h to ablate the MLA with a footprint of 100 × 100 μm^2^, which is clearly not applicable to large-area microlens arrays. Deng et al. [31] attempted to process large areas, but the SEM images showed that the uniformity of array units was not satisfactory.

Grayscale lithography requires only one-time exposure and development to obtain 3D microstructures on photoresist (PR) using grayscale masks. However, the difficulty of producing high-precision masks greatly limits its promotion and application. Nanoimprint technology transfers the microstructure from the mold to the polymer through mechanical hard contact and is classified into hot embossing lithography (HEL) and UV-NIL [32], according to the polymer molding principle used, which are shaped by high temperature and UV light, respectively. This technique is characterized by process simplicity, high efficiency, and good reproducibility, thus enabling its use in mass production. Notably, it requires ultra-precision machining of the opposite target microstructure on a mold first, and as mentioned above, the ultra-precision machining technology is still insufficient for machining large areas. Once, we [33] successfully machined a 6 × 6 microlens array on a silicon substrate using a hot embossing process; however cutting a 600 × 600 μm^2^ high-precision mold already approached our machine tool limit and the surface roughness of the microlens unit was only 17.7 nm, which would affect the light throughput quality of the lens.

Compared with the above technologies, the thermal reflow process provides a viable route for efficiently fabricating large-area microlens arrays. This process forms spherical microlenses with a smooth surface and uniform dimensions based on the liquid surface tension effect [34,35,36,37]. This method adopts common semiconductor processes to simultaneously manufacture all the microlens units, resulting in equally short processing times, even for large-area microstructures. Qiu et al. [38] reflowed photoresist AZ P4620 into a cylindrical microlens array with a surface roughness of 1.66 nm, requiring only heating at 180 °C for 150 s. Extremely low roughness and ultra-smooth surfaces are highly beneficial for improving the light output quality of the lens. However, simply transforming polymers into microlenses, which typically adhere to other substrates, causes these plastic MLAs to be poorly time-stabilized, which greatly limits their application, such as in severe environments characterized by high temperature and vibration. In this paper, we investigated transferring polymer MLAs formed by thermal reflow to rigid substrates underneath, such as silicon, through inductively coupled plasma (ICP) etching. This process ionizes appropriate gases into plasmas, which are sputtered vertically down onto the polymer and substrate, accelerated by an electric field, to achieve large-area etching. Also, during the ICP etching, the final size of the microlens transferred to the substrate can be adjusted by controlling the selectivity to compensate for any deviation of the reflowed structure from the desired dimensions. Experiments were conducted to evaluate the factors affecting selectivity, including RF power and gas flow.

## 2. Materials and Methods

### 2.1. Materials

The 4-inch optical monocrystalline silicon wafers used as the substrate material were customized from Meixin Electronics (Tianjin, China), with N-type doping, a crystal orientation of <111>, and a thickness of 200 ± 15 μm. The silicon wafers were diced into 1-inch samples using a laser ultra-precision processing system (DelphiLaser, UP-D, Suzhou, China). All the samples were ultrasonically cleaned with acetone, isopropyl alcohol, and anhydrous ethanol sequentially for 3 min and were baked at 100 °C for 10 min before the experiment. Photoresists, such as AZ5214 and S1813, and developer NMD (TMAH 2.38%) were sourced from Resemi (Suzhou, China). The NMP solution obtained from Shanghai Aladdin (Shanghai, China) was used to remove any residual photoresist on the wafer surface.

### 2.2. Experiment

**Figure 1:**
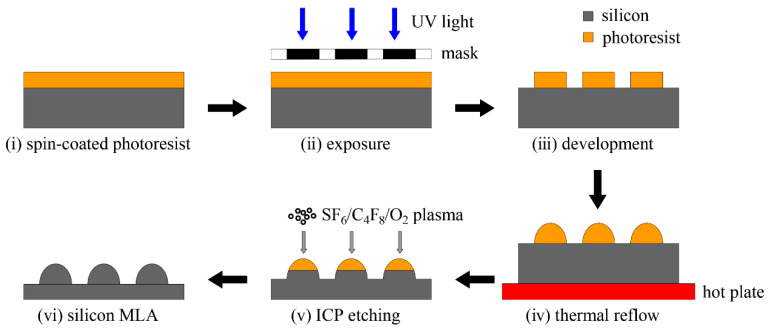
Illustration of thermal reflow and the ICP etching process.

**Figure 2:**
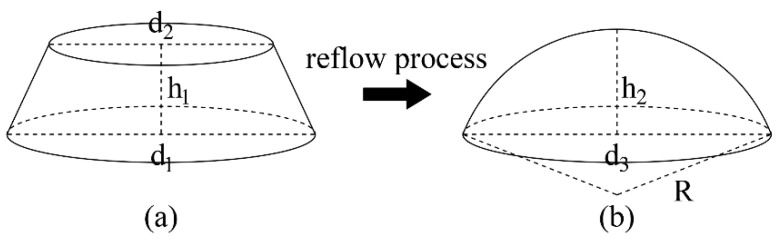
Simplified model before and after reflow. (**a**) The circular truncated cone before reflow and (**b**) the spherical structure after reflow.

The fabrication process for large-area silicon MLAs in this work is depicted in Figure 1. First, the photoresist, including AZ5214 or S1813, was spin-coated on the silicon wafer (step i). The thickness of the film depended on the spin speed and the viscosity of the photoresist. This was followed by prebaking on a hot plate at 100 °C for 60–90 s to ensure the adhesion of the PR layer to the substrate. Next, the exposure process (step ii) was performed using an MA/BA8 lithography machine (SUSS MicroTec, Garching, Germany) and the prescribed intensity of UV light was 23.45 mW/cm^−2^. Since the thickness of the photoresist film was mainly below 3 µm, an exposure time of 7 s was sufficient. We chose soft contact with a photomask as the exposure mode. Although not as effective as non-contact exposures, such as laser direct writing [39], displacement Talbot lithography (DTL) [40] can completely avoid the issue of wafer warpage. The lower contact pressure of the soft contact can maximize the uniformity of the lithography microstructures while ensuring full contact between the photomask and the photoresist layer on the 1-inch silicon substrate. Subsequently, the wafer was immersed in NMD solution for 45 s for development (step iii), thereby removing the photoresist illuminated by the UV light in step ii. After exposure and development, the initial cylinder array was processed on the substrate, but the actual structure was similar to a circular truncated cone due to the imperfect photolithography process, as shown in Figure 2a.

The following reflow process (step iv) entailed heating the wafer with the photoresist cylinder array for enough time on a hot plate, which was set above the glass transition temperature (*T_g_*) of the photoresist. As the temperature rose above *T_g_*, the photoresist behaved as a viscoelastic material, at which time its flowability was enhanced to easily deform the photoresist cylinders into spherical structures (shown in Figure 2b), driven by surface tension. The photoresists used for the experiments were AZ5214 and S1813, with heating temperatures of 180 °C and 150 °C, respectively, for 3 min. The microlens size after reflow was related to the original cylinder thickness and diameter, and the volume may have been reduced due to solvent evaporation during the heating process. Assuming that the volume reduction percentage (the ratio of the volume after and before reflow) is *k*, the following relationship exists for the volume change:

(1) $$k\cdot 2h(d_{1}^{2}+d_{2}^{2}+d_{1}d_{2})=h_{2}(3d_{3}^{2}+4h_{2}^{2})$$

**Figure 3:**
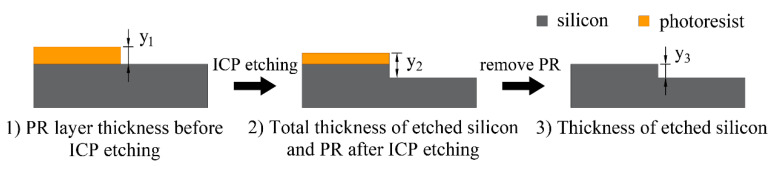
Schematic diagram of the selectivity calculation.

Finally, the target MLA was etched onto silicon through ICP etching (step v), which was performed using the Oxford etching system (Oxford Instruments, Plasma lab system 100 ICP 180, Yatton, UK). The etcher was equipped with two RF sources, both with a frequency of 13.56 Mhz, one of which acted as an ICP generator and was connected to a spiral coil wound outside the chamber to generate an inductively coupled electric field. The other RF source was connected to an electrode below the sample plate inside the chamber, called the CCP generator [41,42]. During ICP etching, selected gases entering the etching chamber generate high density plasma via glow discharge under the effect of the electric field, and the plasma density is affected by the power of the coil (ICP power). Then, the ionized plasma is accelerated by the bias voltage generated by the CCP generator to bombard the wafer downward, thereby removing the surface material physically and chemically [43,44,45,46,47]. We chose SF_6_, C_4_F_8_, and O_2_ to be injected simultaneously as process gases, where SF_6_ and O_2_ were mainly used for etching silicon and the photoresist, respectively. The addition of C_4_F_8_ increased the anisotropy of the etching process, which incidentally slowed down the etching rate. Sometimes the microstructure after reflow may differ from the desired size; fortunately, this error can be corrected by adjusting the etching selectivity (the ratio of the etching rate of silicon to photoresist) to control the finalized structure transferred to silicon. If the reflow size is higher than ideal, a selectivity of less than 1 can be applied to reduce the height. Conversely, if there is an undersized error, a selectivity greater than 1 is required. In order to obtain accurate microlens dimensions on silicon, the influence of RF power and gas flow on etching selectivity under constant ICP power and chamber pressure was investigated. The selectivity *S_R_* is calculated using the following formula (illustrated in Figure 3):

(2) $$S_{R}=\frac{E_{Si}}{E_{PR}}=\frac{y_{3}}{y_{1}-y_{2}+y_{3}}$$

### 2.3. Characterization

The photoresist thickness and step height were measured using a stylus profilometer (Bruker, DektakXT, Billerica, MA, USA), and the surface morphology of the MLA units was observed using a metallographic microscope (Zeiss, Axiocam 208 color, Jena, Germany) and SEM (FEI, Helios 5, Hillsboro, OR, USA). A white light interferometer (Zygo, NewView 9000, Middlefield, CT, USA) and an AFM (Bruker, Demension Icon) were utilized to characterize the surface roughness and 3D profiles of the microstructures. Nine microlens units from the edge and center regions of the processed MLA were selected to calculate the average and uniformity of each parameter, including the diameter, height, and roughness. The uniformity is characterized by the following equation:

(3) $$uniformity=\frac{V_{max}-V_{min}}{2\times V_{average}}\times 100\%$$

where *V_max_* and *V_min_* are the maximum and minimum values measured, respectively, and *V_average_* is the average value of the relevant parameter calculated over all the measured values. From the above equation, a smaller value of uniformity indicates better array consistency.

## 3. Results and Discussion

### 3.1. Results and Analysis of Thermal Reflow

After lithography and the thermal reflow process, photoresist microlenses formed on the silicon substrate. To clarify the effect of the size before reflow on the lens shape, group experiments with different bottom diameters *d*_1_ were designed at spin-coating speeds of 1000 rpm and 1300 rpm. The obtained results are listed in Table 1. The thickness of the PR film was roughly 3 μm at 1000 rpm and 2.6 μm at 1300 rpm. For the reflow experiment, we customized another photomask with multiple 5 × 10 cylinder arrays. The cylinder units of these arrays were of different diameters, but all had a pitch of 100 μm. Through a pre-experiment on reflow time, it was observed that all the photoresist cylinders could reflow into complete spherical structures within 2 min. After 3 min, the reflow tended to stabilize and the microlens shape was basically unchanged. So, we uniformly set the reflow time to 3 min to avoid time interference.

**Figure 4:**
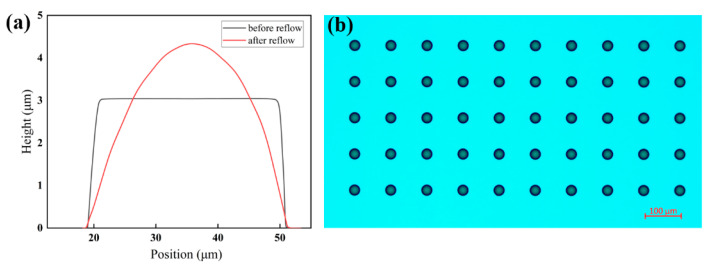
(**a**) Comparison of microlens contour before and after reflow of case 1 in Table 1 and (**b**) the image of the photoresist after reflow.

The section profiles before and after reflow were extracted for comparison using the stylus profilometer, as shown in Figure 4. Combined with *d*_1_ and *d*_3_ in Table 1, it can be seen that the bottom diameter of the photoresist before and after reflow remained almost unchanged, which is due to the fact that the contact surface between the photoresist and substrate was already solidified when the photoresist temperature had not risen above the glass transition temperature, resulting in the reflow being essentially unaffected by the contact angle. This means that controlling the bottom diameter of the photoresist cylinder before reflow can determine the bottom diameter of the microlens after reflow. The cylinder layout was replicated from the mask to the photoresist using lithography. So, when the mask is designed and fabricated according to the target microlens array before the experiments start, this indicates that the bottom aperture of the lens has been defined. From the experimental results of groups 1–4 and 5–8 in Table 1, the volume reduction percentage *k* gradually reduced with an increase in the truncated cone diameter at the same thickness. This was attributed to the larger exposed surface area resulting from increased diameters, which exacerbated the volatilization of the photoresist solvent during heating. The variable *k* makes it difficult to control the lens height *h*_2_, which requires numerous experiments to achieve the desired size. The prolonged time required for thermal reflow poses an unavoidable problem. Fortunately, ICP etching provides a solution by controlling the etching selectivity ratio, which we have presented in the next section.

**Figure 5:**
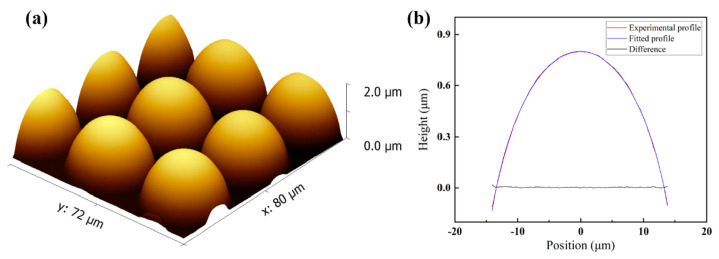
(**a**) The AFM image of the microlens after the reflow process and (**b**) comparisons of the reflowed profile and fitted structure.

The whole wafer was directly placed on the hot plate, and all parts of the wafer were uniformly heated during thermal reflow. Consequently, all the microlens units of the entire array were shaped simultaneously, making the fabrication time independent of the array scale. We successfully used photoresist S1813 to machine a 128 × 128 MLA on a silicon substrate with a microlens unit diameter of 30 μm and a total area of nearly 15 mm^2^. The 3 μm thick cylinder arrays were heated for 3 min and reflowed into hemisphere microlenses with a height of 1.78 μm and a uniformity of 0.02%. A roughness average of 1.13 nm with a 0.50% uniformity was calculated by fitting the extracted microlens profiles through MATLAB R2021a. The 3D morphology in Figure 5 shows the smooth surface of the microlens, attributed to surface tension, and the machining of a larger microstructure area with the same heating time, leading to high process efficiency. There are the two distinguished characteristics of the thermal reflow process.

### 3.2. Results and Analysis of ICP Etching

**Figure 6:**
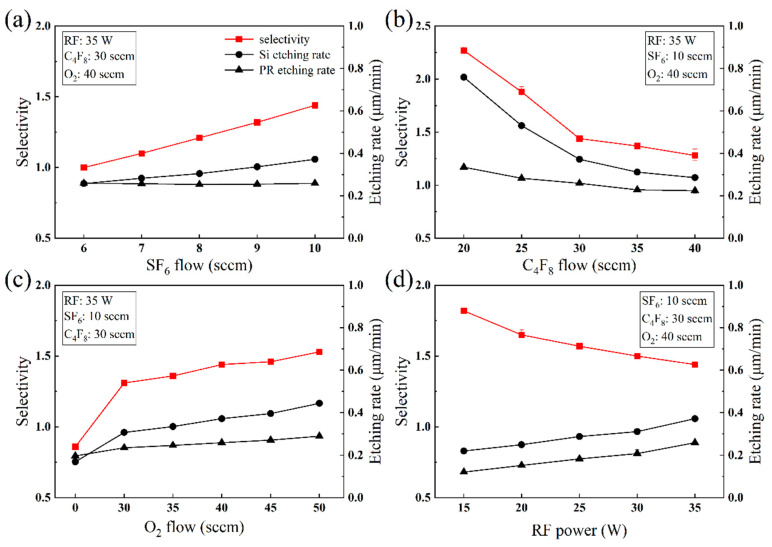
The effect of (**a**) RF power, (**b**) SF_6_, (**c**) C_4_F_8_, and (**d**) O_2_ on etching selectivity.

During ICP etching, the generated plasma etched the photoresist and the silicon substrate concurrently, gradually transferring the microstructure down to the substrate. When the photoresist etching rate was equal to the silicon etching rate, i.e., the etching selectivity *S_R_* = 1, the microstructure on the photoresist was transferred to the silicon at 1:1. Moreover, the final microstructure height decreased when the photoresist etching rate was larger than the silicon etching rate, i.e., *S_R_* < 1, and inversely, it increased at a selectivity of *S_R_* > 1. The *h*_1_ in Table 1 reveals that the photoresist layer thickness may change slightly even at the same spin-coating speed, and the volume reduction percentage *k* also varies with the cylinder diameters and thickness, resulting in the deviation of reflowed spherical microstructures from the expected morphology. This error can then be repaired by adjusting the etching selectivity. The etching results are related to process parameters, such as chamber pressure, ICP power, RF power, gas flow, and etching time, among which ICP power and chamber pressure are responsible for controlling all plasma densities and have a minor influence on the selectivity. Therefore, we kept the ICP power at 800 W and the chamber pressure at 10 mTorr constant in our experiments. Several experiments were performed to research the relationship between the RF power and gas flow of C_4_F_8_, SF_6_, and O_2_ on selectivity. The calculated experimental results are shown in Table 2 and visualized in Figure 6.

**Table 2:** Results of the selectivity adjustment experiments.

Number	RF Power (W)	Gas Flow (sccm)			Height (μm)			Etching Rate (μm/min)		*S_R_*
		SF_6_	C_4_F_8_	O_2_	*y*_1_	*y*_1_	*y*_3_	*E_PR_*	*E_Si_*	
1	35	10	30	0	3.343	3.258	0.507	0.197	0.169	0.86
2	35	10	30	40	2.762	3.101	1.116	0.259	0.372	1.44
3	15	10	30	40	2.861	3.158	0.660	0.121	0.220	1.82
4	20	10	30	40	2.971	3.266	0.749	0.152	0.250	1.65
5	25	10	30	40	2.881	3.195	0.863	0.183	0.288	1.57
6	30	10	30	40	2.822	3.132	0.934	0.208	0.311	1.50
7	35	6	30	40	3.308	3.305	0.774	0.259	0.258	1.00
8	35	7	30	40	3.106	3.183	0.850	0.257	0.283	1.10
9	35	8	30	40	3.467	3.623	0.916	0.253	0.305	1.21
10	35	9	30	40	3.513	3.759	1.012	0.255	0.337	1.32
11	35	10	20	40	3.525	4.797	2.276	0.335	0.759	2.27
12	35	10	25	40	3.559	4.303	1.593	0.283	0.531	1.88
13	35	10	35	40	3.302	3.554	0.935	0.228	0.312	1.37
14	35	10	40	40	3.140	3.326	0.859	0.224	0.286	1.28
15	35	10	30	30	2.966	3.182	0.922	0.235	0.307	1.31
16	35	10	30	35	2.926	3.192	1.004	0.246	0.335	1.36
17	35	10	30	45	2.950	3.325	1.188	0.271	0.396	1.46
18	35	10	30	50	2.901	3.364	1.331	0.290	0.444	1.53

Figure 6 intuitively shows that the selectivity increases with SF_6_ and O_2_ flow and decreases with RF power and C_4_F_8_ flow. During the ICP etching process, C_4_F_8_ ionizes and deposits on the exposed surface, forming a fluorocarbon polymer that prevents etching, called passivation, and SF_6_ and O_2_ ionize into plasma for etching the silicon and photoresist. The etching ions can only contact and remove the silicon and photoresist materials after consuming the passivation layer deposited on their surfaces. So, an increase in C_4_F_8_ flow enhances the protective action, causing degradation of the etching rate of both silicon and photoresist, as shown in Figure 6a. Because the area of silicon in the etching region is larger than the area of photoresist, C_4_F_8_ is more influential on the silicon etching rate, resulting in decreased selectivity. The F ions ionized from SF_6_ are mainly employed for etching silicon, so when SF_6_ flow increases, the F ion density in the plasma becomes higher, which improves the etching rate of silicon. The photoresist etching rate remains almost static in Figure 6b, and the selectivity rises as a result. O_2_ is used to enhance the etching of polymer materials, but Figure 6c exhibits that the addition of O_2_ changes the selectivity from smaller than 1 to greater than 1. This suggests that O_2_ reacts with the C atoms of C_4_F_8_, weakening the passivation of C_4_F_8_ and leading to an increase in the silicon etching rate. RF power boosts the kinetic energy of the plasma bombarding material, which enhances the physical etching. It can be seen from Figure 6d that the etching rates of both silicon and photoresist rose with increasing RF power. These experimental results demonstrate that RF power and gas flow can regularly change the selectivity, but this does not mean that these process parameters are appropriate for all situations. When the selectivity needs to be greatly adjusted, SF_6_ is better suited for the task. Partly because SF_6_ only changed the etching rate of silicon, it had almost no impact on the photoresist, making selectivity easily predictable. Additionally, a small variation in SF_6_ flow caused a relatively large change in selectivity, making it convenient for rapid and extensive adjustments. O_2_ is recommended for minor selectivity adjustment because a 20 sccm change in O_2_ flow only changed the selectivity by 0.21, as shown in Table 2. It must be noted that changing the etching parameters also brings some side effects; thus, optimizing the process recipe requires caution. For example, an increase in SF_6_ gas flow will enhance the isotropy of the etching process, whereas a larger RF power makes the process more anisotropic.

**Figure 7:**
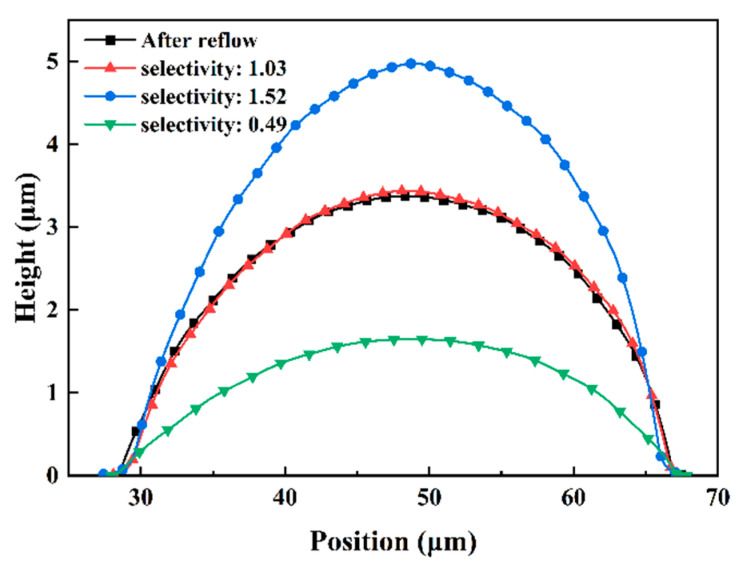
Comparison of profiles with different etching selectivities.

**Figure 8:**
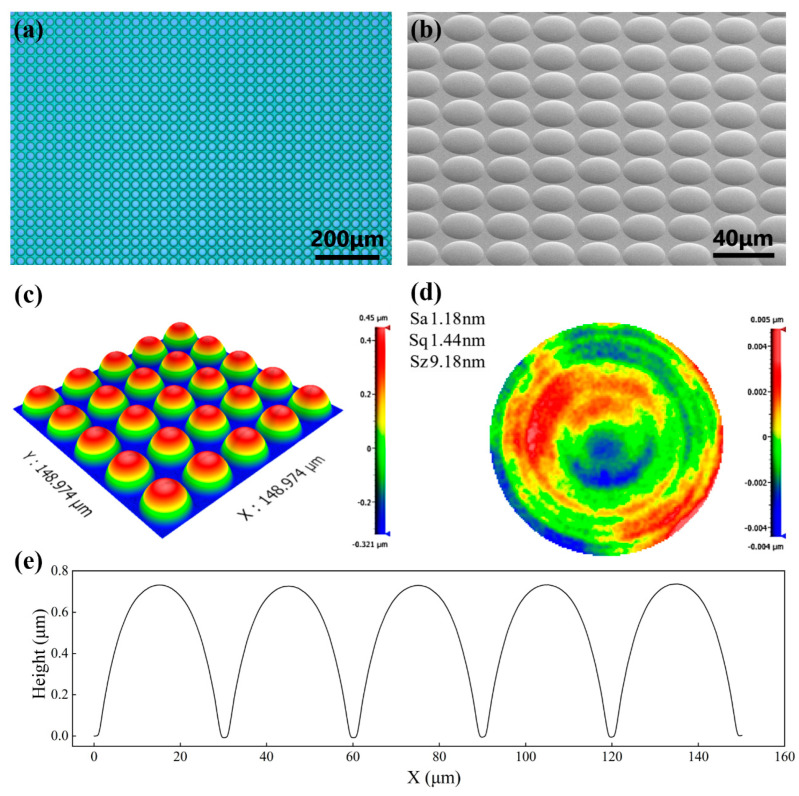
The characterization images of silicon MLA samples with a selectivity of 0.41. (**a**) An image of the 128 × 128 array observed using metallurgical microscopy at 10× magnification, (**b**) SEM image of the MLA, (**c**) 3D morphology of the MLA, (**d**) the roughness inspection image of a microlens unit, and (**e**) the cross-sectional profile of the array.

The present selectivity can be controlled from 0.4 to 2.3, adequately satisfying the etching requirements for most conditions. The same reflowed microlens with different etching selectivities can obtain microstructures with different characterization parameters, including the radius of curvature, which is exhibited in Figure 7. The height of the reflowed microlens before ICP etching was 3.38 μm and the radius of curvature was 60.80 μm. When the selectivity was 1.03, the size of lens on silicon changed slightly. However, the lens height was reduced and the radius of curvature was 118.85 μm when the selectivity was *S_R_* = 0.89. Drastically varied selectivity may transform the lens spherical structure to an ellipsoidal shape, such as 1.52 selectivity in Figure 7, which fits to the major semiaxis a = 21.30 and the minor semiaxis b = 8.62. The 128 × 128 silicon microlens array after 15 min of ICP etching, with a selectivity of 0.41, is presented in Figure 8, with an average surface roughness of 1.2 nm and a uniformity of 0.85%, which confirms the technological feasibility of the thermal reflow process combined with ICP etching for machining large-scale MLAs. 

## 4. Conclusions

In conclusion, thermal reflow processes combined with ICP etching proved to be an efficient fabrication process for machining large-scale microlens arrays. The cylinder array after lithography was reflowed into smooth spherical microstructures using reflow processes driven by surface tension, which were subsequently delivered to the silicon substrate by ICP etching. We discovered that the bottom diameter of the microlens remained invariant because the contact surface of photoresist and substrate had already cured under the influence of high temperature before the reflow began. This characteristic makes it convenient for controlling the microlens dimensions. Although the photoresist volume reduction percentage varied with the thickness of the cylinder, resulting in a deviation of the size after reflow from the designed size, this can be rectified by adjusting the etching selectivity. The selectivity increased with O_2_ and SF_6_ flow, with an opposite contribution of C_4_F_8_ and RF power. A 128 × 128 silicon microlens array with good uniformity and a low surface roughness of 1.2 nm was fabricated in a short period of time, illustrating the excellence of the thermal reflow process for mass production. Our future work includes fabricating larger-scale microlens arrays and constructing optical platforms for measuring the optical performance of MLAs.

## Figures and Tables

**Table 1:** Experimental data and the results of the thermal reflow process.

Number	Cylinder before Reflow (μm)			Microlens after Reflow (μm)			*k*
	*d*_1_	*d*_2_	*h*_1_	*d*_3_	*h*_2_	*R*	
1	32.65	28.08	3.03	32.88	4.31	33.51	0.850
2	37.50	33.48	3.00	38.28	4.27	45.03	0.841
3	42.73	38.18	3.00	42.53	4.27	55.06	0.797
4	47.73	43.08	2.98	47.50	4.09	70.92	0.758
5	32.60	28.55	2.65	32.33	3.75	36.88	0.804
6	36.20	33.85	2.63	36.45	3.71	46.60	0.774
7	42.13	38.45	2.62	42.40	3.53	65.39	0.753
8	46.95	43.53	2.61	47.35	3.36	85.11	0.709

## Data Availability

The original contributions presented in the study are included in the article, further inquiries can be directed to the corresponding authors.
